# Mr. Mill’s Case of Diarrhœa from Ulceration of the Intestinal Glands Cured by the Internal Use of the Chloride of Sodium

**Published:** 1842-05-07

**Authors:** C. J. Mill

**Affiliations:** Surgeon, Kirriemuir


*Case in which diarrhœa from ulceration of intestinal glands disappeared under the internal use of chloride of sodium.* By Mr. C. J. Mill, Surgeon, Kirriemuir.—A. F., aged 29, after exposure to cold and wet, was seized with rigors followed by acute pain in the head and abdomen, and violent purging. He stated that for these symptoms he had lost a few ounces of blood, and taken calomel and salts. I saw him after he had been a fortnight confined to bed, and found him then very much reduced, almost unable to sit up in bed; with pulse at 90, full and hard; the abdomen tender on pressure or motion; purging almost incessant; matters passed sometimes bloody mucus in large quantities, at other times very dark thin feculent matter, like fluid pitch, always excessively fœtid. He complained much of tormina; also of irritation of urinary organs. He had great thirst; the tongue was glazed, red, and dry; total loathing of food. He complained still of pain in the head; and last night he was a little incoherent.

I opened a vein and bled him to approaching syncope; gave calomel, castor oil, and opium; had the head shaved, and cold lotions applied; blistered the abdomen, and then applied hot poultices. Arrow-root gruel was prescribed for food.

After bleeding, &c. the pain in abdomen was much relieved, but the purging continued unabated. The matters discharged were still as dark coloured (not such a colour as arises from the use of mercurials,) and fœtid as ever. The tongue and teeth were covered with black sordes; the pulse was quick, small and weak; the patient had low muttering delirium; but the pain in the abdomen was gone. As the purging was giving so very great uneasiness to the patient whom I considered sinking from sphacelus, the result of neglected inflammation of the mucous membrane of the bowels during typhoid fever, I ordered chalk mixture to be given every three hours until it should abate; and gave wine and nutritious soups. These measures were followed by relief at the time; but whenever the chalk mixture was intermitted, the tormina and purging recurred as before.

The fœtor from the evacuations and breath was now so excessive that I gave some solution of chloride of sodium to sprinkle in and about the patient's bed to quench the smell. It occurred to me, that, if given internally, it might act in a similar way by correcting fœtor, and inducing healthy action. I therefore ordered a drachm of it to be given in half an ounce of chalk mixture, and repeated every four hours. Next day when I visited my patient, I was quite delighted and surprised to find his appearance so much changed for the better. He had had a refreshing night's sleep; the purging was abated; the tormina ceased; there was no fœtor; and the black crust was separating at the edge of the tongue. The countenance was expressive of hope. He had taken a little arrow-root in the morning. I ordered the chloride to be continued, omitting the chalk mixture, and substituting wine in its place. His recovery has been rapid, and is now complete; only requiring an occasional small dose of castor oil.—*Ibid.*

